# Assessing the Adhesiveness and Long-Term Behaviour of Piezoresistive Strain Sensor Materials for Application in Structural Health Monitored Structures

**DOI:** 10.3390/s25061659

**Published:** 2025-03-07

**Authors:** Daniel Kimpfbeck, Herbert Enser, Jonas Wagner, Lukas Heinzlmeier, Boris Buchroithner, Pavel Kulha, Bettina Heise, Günther Hannesschläger, Christoph Kralovec, Martin Schagerl

**Affiliations:** 1 Institute of Structural Lightweight Design, Johannes Kepler University, 4040 Linz, Austria; jonas.wagner@jku.at (J.W.); lukas.heinzlmeier@jku.at (L.H.); christoph.kralovec@jku.at (C.K.); martin.schagerl@jku.at (M.S.); 2 Institute for Microelectronics and Microsensors, Johannes Kepler University, 4040 Linz, Austria; 3 PROFACTOR GmbH, 4407 Steyr, Austria; boris.buchroithner@profactor.at (B.B.); pavel.kulha@profactor.at (P.K.); 4 Research Center for Non-Destructive Testing (RECENDT)-GmbH, 4040 Linz, Austria; bettina.heise@recendt.at (B.H.); guenther.hannesschlaeger@recendt.at (G.H.)

**Keywords:** carbon nanotubes, carbon black, PEDOT:PSS, strain sensor, piezoresistivity, durability, adhesion, structural health monitoring, spraying, inkjet printing, screen printing

## Abstract

The durability of piezoresistive sensor materials is a core prerequisite for their implementation in structural health monitoring systems. In this work, three piezoresistive materials were subjected to extensive cyclic tensile loadings, and their behaviour was analysed before, after, and during testing. To this end, aluminium specimens were coated with three different industry-grade lacquers, and then piezoresistive materials were applied onto each specimen. Sensors made from carbon black displayed excellent linearity even after tensile loading cycles ($R^{2}>0.88$). A decline in linearity of all sensors based on carbon allotropes was discovered, whereas the polymer-based sensors improved. Furthermore, their adhesion to the substrate is of great importance. Good adhesion ensures the strains in the underlying structure are correctly transmitted into the sensor materials. Based on contact angle measurements of liquids on sensor materials and on lacquers, their work of adhesion was determined. The findings were verified by tape adhesion tests.

## 1. Introduction

Structural health monitoring (SHM) by strain mapping is considered to be the most promising approach in strain-based structural health monitoring [1]. Different approaches to strain sensing material exist, from traditional strain gauges and fibre-optic sensors to self-sensing approaches. Whilst they represent well-established systems, one major drawback associated with the former two technologies is that they detect strain only locally. By using conducting materials dispersed in solutions, large areas can be covered with piezoresistive thin films, and by that, problems that arise with the need for a dense distribution of single sensors can be overcome (e.g., cable management). The advantages of self-sensing SHM are that the structure itself acts as the sensor, which means that no labour-intensive sensor application is necessary. Also, the sensor cannot fail without the structure itself failing. If the sensor fails, there is a high likelihood that the structure itself has failed as well. However, due to the excellent electrical conductivity of metallic structures, electric resistance changes caused by deformations are small and thus difficult to detect. Utilising self-sensing SHM on carbon-fibre-reinforced plastics poses the same problem, with the additional difficulty of having to handle an anisotropic material. Applying inks containing conductive materials onto structures generally results in isotropic sensors which may be tuned to be anisotropic, if desired [2]. Furthermore, by using thin-film sensors, even non-conductive structures may be monitored.

The range of application is manifold. Current generations of passenger aircraft comprise up to 50% fibre-reinforced plastics. Many important primary load-bearing elements, such as push–pull rods or struts, are still made from aluminium. Although the materials’ long-term durability is well understood, SHM could help further reduce weight, thus enhancing performance whilst also increasing safety by allowing the continuous on-line monitoring of the load-bearing structure and thus assessing its remaining structural health [3]. One simple and cost-effective method is the application of piezoresistive thin-film sensors directly onto the surface of the structure, either locally or over large areas. But reliability issues such as short circuits or disbonding from the structure still remain.

A multitude of conductive materials have successfully been utilised for the fabrication of spatial strain sensors. These include allotropes of carbon, such as carbon black (CB) [4,5,6] or carbon nanotubes (CNTs) [7,8], and conductive polymers like polyaniline [9,10], ionic liquids [11,12], or poly(3,4-ethylenedioxythiophene) (PEDOT) [13,14,15], often in combination with polystyrene sulphonate (PSS). Carbon nanomaterials, especially carbon nanotubes, have been a staple in spatial strain sensor design for the last few decades. This popularity can be attributed to their excellent electrical and thermal conductivity, extraordinary mechanical strength, a high aspect ratio, ease of application, and in the case of CB, low cost [6,16,17]. Similarly, PEDOT:PSS is considered as one of the most popular conductive polymer for strain monitoring. It is highly conductive, dispersible in water, and easier to process than other conductive polymers [18]. When these materials are applied to a surface, changes in electrical resistance as a response to mechanical strain can directly be measured. This is called the piezoresistive effect; therefore, the materials act as piezoresistive sensors.

In this article, we investigate the strain sensing behaviour of piezoresistive thin-film sensors applied to coated aluminium specimens for their continuous and long-term use in structural health monitoring applications. Conductive materials have been processed by airbrush spraying [19], inkjet printing [20], and screen printing [4]; thus, they were utilised here. Aluminium specimens are coated with assorted lacquers to determine the sensor materials’ suitability for structural health monitoring applications and the finishes’ influence on sensor performance. These lacquers are generally applied as a protective coating in harsh environments, but here, they serve the additional purpose of an insulating layer, allowing the use of piezoresistive thin-film sensors in the first place. Proper selection of lacquer is an important subject matter since they are generally specific to the application and the substrate. If sensor materials for strain sensing work well regardless of the lacquer system they were applied to, it would allow for an unconstrained selection of lacquer tailored only to specific substrate materials.

This article aims to provide a comparative study of different popular strain sensing materials regarding their adhesiveness to substrates and the effects of long-term cyclic testing on their strain sensing capabilities. To the knowledge of the authors, the behaviour of the sensor materials presented here has not yet been the subject of a comparative study involving such an extensive number of loading cycles. Different scalable application methods are utilised that have the potential of offering either global or local strain monitoring capabilities that may be used to inspect relatively large areas. The versatility of the sensor design enables simple resistance measurement of the sensor or spatial load monitoring over the entire sensor surface using, e.g., electrical impedance tomography [21]. The sensor materials are applied to different lacquer systems, and their compatibility is evaluated by their adherence, which is one of the main parameters used when assessing the quality of a print [22]. The compatibility of the sensor material with the substrate is important for real-life applications.

## 2. Materials and Methods

### 2.1. Specimen Layout

**Figure 1:**
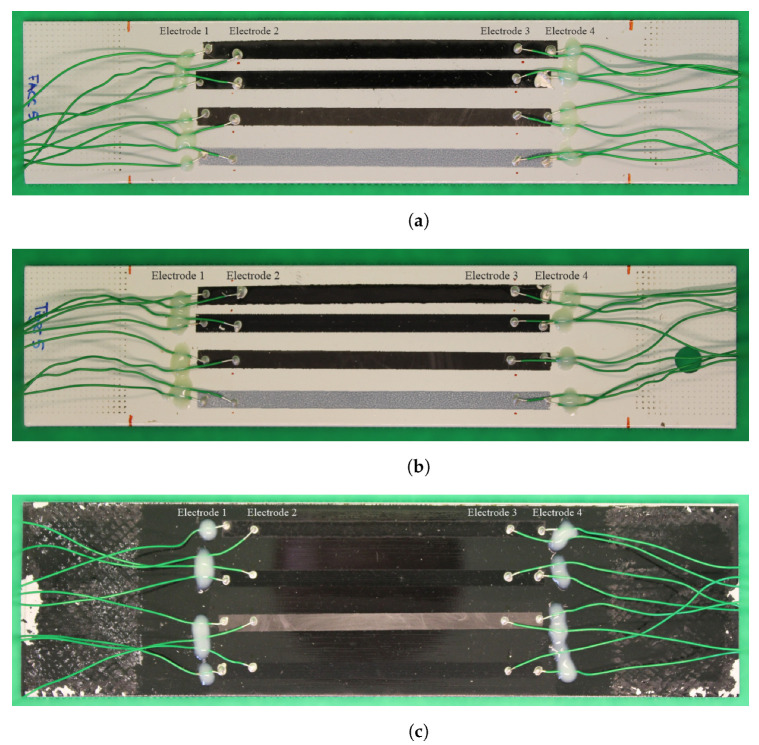
Coated specimen with all sensor materials applied and probes attached for 4-point probe measurement after fatigue loading, (**a**) FACC, (**b**) TIGER, (**c**) PRO. Sensor materials from top: airbrush-sprayed CNT, inkjet-printed CNT, CB, and PEDOT:PSS.

The specimens in this study are made of aluminium, each with one of the three lacquer systems applied to them. These lacquer systems are typically used as a coating to protect the substrate from harsh environmental conditions. However, in this case, they serve as an insulation, allowing the application of sensor materials and measuring their change in resistance without creating a short circuit. Each specimen in this study contains all three sensor materials (CNT, CB and PEDOT:PSS) side by side. CNTs were applied in two different ways (airbrush spraying and inkjet printing); thus, a total of four individual sensors exist on each specimen. The dimensions are the same for all sensors, namely, 100 × 5 mm^2^. They were applied in parallel with each other and oriented in the loading direction. Their placement guarantees similar strain states prevailing in all sensors at the same time during loading, resulting in each experiencing the same amount of wear. Each of the sensors is contacted by four electrodes in line to enable 4-point probe measurements while mechanical loading is applied to the specimen. The cables that lead to the instruments are secured by a stress relief. The three specimens including cabling are depicted in Figure 1.

### 2.2. Specimen Preparation

The substrates that served as the basis for this study are aluminium specimens cut from larger plates, sanded with 2000 grid sandpaper, and subsequently cleaned with isopropyl alcohol and acetone. Specimens were then given to FACC AG, an Austrian supplier of lightweight structural components of aviation situated in Ried im Innkreis, Austria, TIGER Coatings GmbH & Co. KG, an Austrian manufacturer of multiple coating solutions, situated in Wels, Austria, and PROFACTOR GmbH, a research institute located in Steyr in Upper Austria, to apply coatings.

The lacquer applied by FACC AG (Ried im Innkreis, Austria) is a PPG Aerospace Desothane HS CA8800 (Pittsburgh, PA, USA) and was applied in a wet condition. The topcoat is polyurethane-based. Specimens painted with this lacquer are referred to as FACC.

In contrast, coating applied by TIGER Coatings GmbH & Co. KG (Wels, Austria) is a TIGER Drylac^®^59/10362 RAL 9010 and was applied by powder coating and is based on polyester. Specimens painted with this coat are referred to as TIGER. Lacquers by FACC and TIGER were chosen because they are used as coating for exterior applications.

The lacquer applied by PROFACTOR GmbH (Steyr, Austria)was an acrylate-based TIGER TIGITAL Series 150/3.1 K (Product ID: 150/31801) and was applied by inkjet printing. A multi-material inkjet printer Polyjet™Connex2 from Stratasys (Rehovot, Israel) was used to deposit the lacquer. One pinned and two spreading layers form a smooth surface that was subsequently fully cured in a UV oven according to manufacturer specifications. Before inkjet printing, the sample surface was treated with the PROFACTOR adhesion promoter HMNP-12. For this, the sample was cleaned with Ethanol, dried, and oxygen plasma-activated. Thereafter, HMNP-12 was spray-coated on the surface and baked at 120 °C for 2 min. Specimens painted with this lacquer are referred to as PRO.

After the specimens were lacquered, carbon black Paste C2130819D1 by SunChemical (Parsippany-Troy Hills, NJ, USA) and PEDOT:PSS CLEVIOS S V4 by Heraeus (Hanau, Germany) were applied by screen printing using a semi-automatic RokuPrint SD 05 (Oberdischingen, Germany). First, CB was applied and the specimen was cured at 60 °C for 30 min. According to the datasheet, the solid content is between 37 and 41% when cured at 130 °C; thus, it is assumed that it is at or marginally lower than 37% in this particular example. No further information on the pastes’ composition is given by the manufacturer. Afterwards, PEDOT:PSS was applied and cured at 130 °C for 15 min. A second layer of PEDOT:PSS was applied thereafter on top of the first one because it was barely visible, and thus, contacting for electrical measurements could prove to be difficult. The same printing speed and 120T mesh were used for all prints.

After screen printing was completed, self-made CNT ink was airbrushed onto the specimen using an Evolution airbrush by Harder & Steenbeck GmbH & Co. KG (Norderstedt, Germany). The ink was prepared by solution mixing and contains 0.3 wt.% multi-walled carbon nanotubes purchased from Sigma-Aldrich. (St. Louis, MO, USA) Pluronic F-127, also acquired from Sigma-Aldrich, was used as a surfactant. For details on ink fabrication, see [23]. Stencils were cut from self-adhering plastic sheets by laser cutting and attached to the specimen. Application was as dry as possible to minimise the coffee ring effect, meaning the airbrush was set up to generate fine ink droplets that would dry instantly upon contact with the substrate. A total of 5 layers were applied. Lastly, CNT ink was applied by inkjet printing. The process was also described before in [23]. Between 4 and 5 layers were applied, depending on resulting opaqueness. During the process, the substrate was heated to 60 °C by a hot plate to ensure quick evaporation of water. Omission of heating would lead to uneven CNT distribution due to the coffee ring effect. Application of CNT sensors was not followed by a curing step. The same CNT ink was used for airbrush deposition and inkjet printing. The content of CNTs in the ink was chosen to avoid clogging of the inkjet printers’ nozzle. To measure a sensor’s resistivity, it was contacted for 4-point probe measurements using EPO-TEK H20E (Billerica, MA, USA) electrically conductive silver epoxy. Four probes were aligned in one line on each sensor. The distance between the outer and the inner electrodes is between 7 and 10 mm, and the distance between the two inner probes is between 77 and 80 mm. After the application of the silver epoxy, samples were cured for 3 h at 80 °C.

### 2.3. Assessing the Adhesiveness of Sensor Materials on Lacquered Substrates

To evaluate wettability and adhesiveness, contact angle measurements and work of adhesion provide state-of-the-art measures [24,25]. A Krüss DSA 100 (Hamburg, Germany) was utilised for contact angle measurement with water, diiodo-methane, and ethylene glycol. To identify the total surface free energy and its disperse and polar components, a regression analysis was performed according to the Owens–Wendt–Rabel–Kaelble method. Subsequently, the work of adhesion was derived using Equation (1) to quantify the adherence of sensor materials onto the surface.

(1) $$w_{a}=w_{a}^{d}+w_{a}^{p}=2\left(\sqrt{\sigma_{s}^{d}\sigma_{l}^{d}}+\sqrt{\sigma_{s}^{p}\sigma_{l}^{p}}\right),$$

where $w_{a}$ is the work of adhesion, σ is the surface free energy derived from the contact angle measurements, and the indexes *l*, *s*, *d*, and *p* denote liquid, solid, disperse, and polar, respectively [26].

The adhesion of sensor materials on lacquers was further evaluated by peel-off testing. A strip of tesa Tixo 56002 tape (Vienna, Austria) was applied to the sensors and peeled off by hand. Visual inspection of the tape and substrate were made to validate the results from contact angle measurement. Results can be seen in Section 3.1.

### 2.4. Sensor Material Distribution

To investigate the distribution of sensor material on the substrate, 2-point probe resistance measurements were conducted over length *L* of the sensor using a multimeter. Initially, the two probes were positioned on the opposing ends of the sensor. One of the probes was then successively moved inwards in 5 mm increments, and reduced to 1 mm for the last 10 mm. At each step, the resistance was measured; thus, the relationship between length of the sensor and its width *W* became increasingly smaller. The specimens remained in an unstrained state during the procedure. Resistivity ρ was calculated using Equation (2)

(2) $$\rho =\frac{R}{L}\times t\times W,$$

where *R* is the measured resistance, and *t* is the sensor’s thickness.

The degree of coverage is also investigated by optical coherence tomography (OCT). OCT is a non-invasive, non-destructive imaging technique based on the physical phenomenon of low-coherence interferometry and detects small refractive index differences within the sample [27]. The sensor materials interact differently with light than the underlying lacquer, thus allowing the evaluation of sensor material distribution based on scattering, reflection, and absorption features, partly visible in OCT cross-sectional scans. The different intensities of the reflection can be seen from the periodic signals in depth direction, coming from a saturation of the camera. A strong periodic reflection indicates the absence of sensor material. Measurements were taken at a central wavelength of 830 nm across the width of sensors on each surface. Results can be seen in Section 3.2.

### 2.5. Strain Sensing Characterisation

For the characterisation of the sensor material’s strain sensing capabilities, conductivity was measured in a 4-point probe setup. This setup was chosen because it minimises the current flowing through the inner two electrodes where the voltage is measured, and therefore, measurement errors such as contact resistance are reduced. These measurements were taken during the quasi-static and fatigue loading blocks described below. Four electrodes were applied to the sensors in a configuration that enables the majority of the sensor to be within the two innermost probes. Current of $3\times 10^{-4}$ mA was injected into the sensors via the outer two electrodes by using a Keithley 6220 DC (Cleveland, OH, USA) current source. The resulting voltage was measured between the inner two electrodes using an HBM QuantumX MX840B multichannel data acquisition system.

Resistivity ρ was subsequently calculated using Equation (2), where $R=\frac{V}{I}$ was derived from the measured voltage *V* and the applied current *I*, and *L* is the distance between the two innermost electrodes.

An HBM QuantumX MX840B amplifier (Darmstadt, Germany) was used to measure and record the resulting voltage on the inner two electrodes of the sensors at 300 Hz. Electrode spacing and sensor widths were determined with an Olympus SZX10 stereo microscope (Tokyo, Japan). A set of mechanical relays by Shenzhen ELEGOO Technology (Hongkong, China) driven by an Arduino Nano was cycling through the specimens every 40 s, resulting in 10 s long voltage responses to the current excitement of the respective sensor. This setup was necessary because individual sensor’s resistance values were too high for direct measurement with an HBM QuantumX, and only one high-precision DC current source was available. However, the duration of the complete cyclic test is much longer than the measurement time of each sensor, so a continuous measurement and characterisation of each sensor material can be assumed throughout the experiments.

A 37.5 Hz low-pass filter was implemented on raw voltage data from cyclic testing to eliminate high-frequency noise. Then, an LOESS (locally estimated scatterplot smoothing) operation was performed afterwards to further remove noise from the signal. These operations were the same for all voltage data.

For the remainder of this article, when referring to resistivity, changing sensor dimensions are not taken into consideration unless specified. This includes elongation of the substrate during tensile loading and compression normal to the direction of loading due to the Poisson effect. Changes in resistivity therefore result solely from the individual elastoresistive behaviour of each sensor and are a cause of the applied strain.

Specimens were subject to two quasi-static and one fatigue loading block. One quasi-static block was situated before the fatigue block, and another one afterwards. Specimens were loaded up to a fixed load within the substrate material’s linear-elastic limit for 50 cycles at a frequency of 0.2 Hz during the quasi-static loading blocks, and at 10 Hz for $10^{6}$ cycles during the fatigue loading block. These first two loading regimes were implemented to provide a closer look at the behaviour of each sensor material when loaded, and to better detect certain effects like the set-in effect. They also allowed us to investigate a possible change in sensitivity to strain caused by the material’s deterioration as a result of fatigue loading. A cyclic load with a stress ratio of R=0.1, meaning pure tensile loading, was applied to the specimens. The low strain rate during quasi-static testing allowed loading via a linear saw-tooth pattern, whereas for fatigue testing, a sinusoidal wave pattern had to be used. Load levels were the same for both loading blocks.

Experimental loading was conducted by a servo-hydraulic cylinder from Zwick–Roell with a nominal force of 25 kN. To minimise the influence of the aluminium sample, it was loaded in the linear-elastic range. FACC and TIGER specimens were each loaded up to 5400 N, resulting in a strain of $1000\times 10^{-6}$. PRO specimens were larger in width and thickness and therefore loaded to 15,400 N, resulting in strains of up to $1400\times 10^{-6}$. Specimens are kept in place with hydraulic wedge grips by MTS.

For the measurement of surface strain on the specimens, a Digital Image Correlation system by Correlated Solutions, Inc. (Irmo, SC, USA) was positioned facing the specimens during quasi-static testing and recorded at 5 Hz. Strain measurements were taken every 500 cycles at peak- and valley-load during fatigue testing.

Temperature and humidity are known to have an influence on the sensor materials utilised in this article [28,29,30,31,32]. Thus, ambient temperature and relative humidity were recorded in immediate vicinity of the specimens under load throughout the duration of testing.

**Figure 2:**
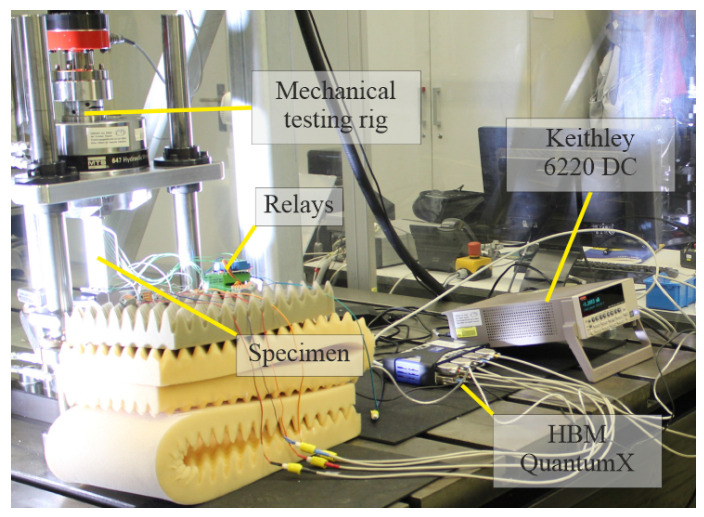
Mechanical testing and data acquisition setup, including aforementioned components.

**Figure 3:**
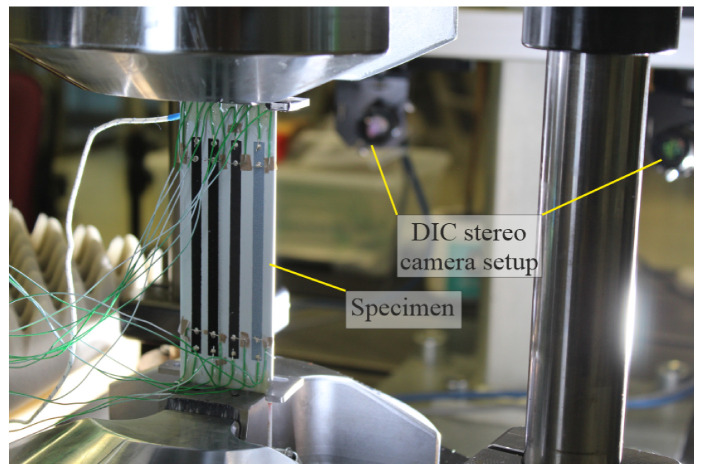
Clamped specimen with stereo cameras used for 3D-DIC evaluation (in the background).

Figure 2 and Figure 3 display the experimental setup for tensile testing.

## 3. Results

### 3.1. Adhesive Properties

**Figure 4:**
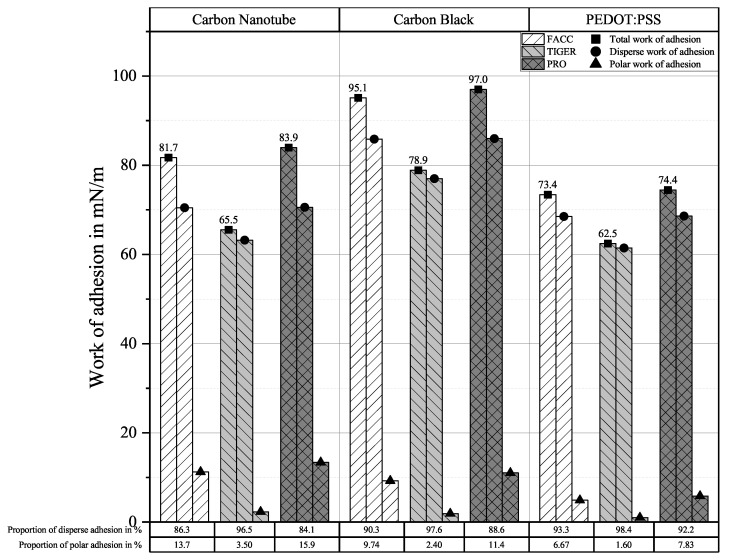
Work of adhesion of sensor materials on substrates derived from contact angle measurements.

Translating surface free energy from contact angle measurements into work of adhesion using Equation (1) allows the identification of the strongest-adhering couple. Due to the almost identical contact angles measured for CNTs independent of the substrate, a mean value was calculated and used for subsequent analyses. Results can be seen in Figure 4.

Carbon nanotube and carbon black sensors show relatively good adhesion on FACC and PRO substrates compared to PEDOT:PSS, with the majority of adhesion coming from disperse interactions. Adhesion to the TIGER substrate is generally weaker than on the other substrates, but the proportion of disperse adhesion is larger. PEDOT:PSS shows the same adhesive tendency regarding substrate influence, but with overall lower total work of adhesion. The disperse proportion is even larger than for the carbon allotrope-based sensors.

Sufficient adhesion between sensor materials and lacquers was discovered for all material combinations by tape adhesion tests. Although residuals of carbon nanotubes and carbon black were discovered on the tape after the peel-off, these residuals detached only from the top of the sensors, and no blank spaces of lacquer were left behind.

**Figure 5:**
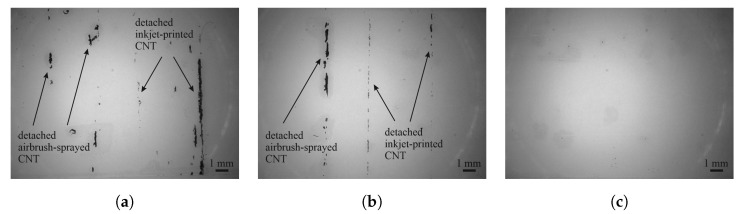
Microscopy images of the adhesive tape after tape adhesion test on carbon nanotube sensors on the (**a**) FACC, (**b**) TIGER, and (**c**) PRO substrates.

In the case of carbon nanotubes, residuals only detached from the sides of the sensor, as can be seen in Figure 5. These outer areas of a printed structure are usually thicker than the inner part due to the coffee ring effect. Slightly more carbon nanotubes detached from the FACC specimen than from TIGER, and no material at all detached from PRO, indicating excellent adhesion.

**Figure 6:**
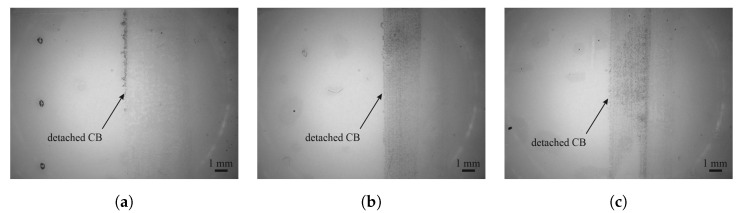
Microscopy images of the adhesive tape after tape adhesion test on CB and PEDOT:PSS on the (**a**) FACC, (**b**) TIGER, and (**c**) PRO substrates.

Figure 6 shows that, in the case of carbon black, a thin layer was peeled off by the adhesive tape with most of the material remaining on the sensor. Adhesion between carbon black and the substrate can be evaluated as good since no larger pieces have come loose. But because carbon black consists of small pieces of graphene that move against each other, the top layer of the sensor can be easily peeled off using the adhesive film.

No detachment of PEDOT:PSS is detectable on any of the tapes; detachments should be visible to the left of CB in Figure 6. This is a result of the high transmittance of PEDOT:PSS, which is around 86 % [33]. The low opaqueness makes it difficult to evaluate adhesion based on residuals on the tape, but work of adhesion data indicate that adhesion between PEDOT:PSS and lacquers should be a little lower than between carbon nanotubes and lacquers.

### 3.2. Sensor Material Distribution

**Figure 7:**
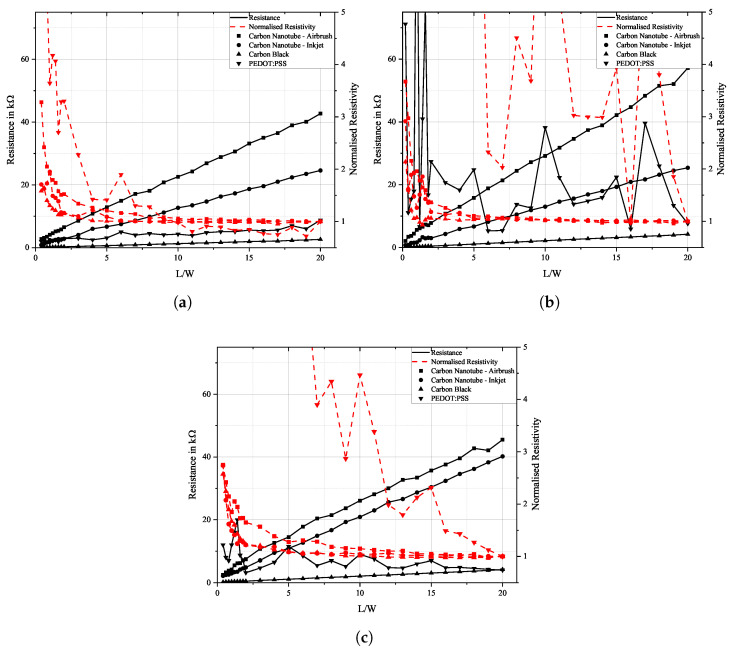
Plot of the change in resistance and resistivity as a function of sensor length L to width W for airbrush-sprayed carbon nanotubes, inkjet-printed carbon nanotubes, carbon black, and PEDOT:PSS on (**a**) FACC, (**b**) TIGER, and (**c**) PRO.

Figure 7 illustrates the measured data from two-point probe resistance measurements. Change in resistance with respect to the sensor’s width-to-length ratio is displayed on the left axis. The length *L* of the sensor was steadily reduced, while the sensor’s width *W* and thickness *t* remained unchanged. Resistance was measured with a digital multimeter Keithley 2110 5 1/2 and resistivity was subsequently calculated using Equation (2) and normalised to the full sensor length.

The measured data show that for ratios of L/W larger than 5, resistance changes linearly with the sensor’s size while resistivity remains constant. This suggests that conductivity is independent of the sensor’s size, and conductive particles are evenly distributed across the sensor. While this is true for carbon allotrope-based sensors, resistance measurements of PEDOT:PSS show a certain non-linearity. On the FACC lacquer, it still declines steadily, but on the other substrates, it shows a weak increase in resistance. This might be caused by the existence of voids in the PEDOT:PSS sensors, which is especially pronounced on the TIGER specimens, which in turn may be traced back to bad wettability and thus inhomogeneous material distribution. Measurements are especially non-linear when L/W becomes smaller than 4, and only a small amount of individual conductive paths remain between the electrodes.

**Figure 8:**
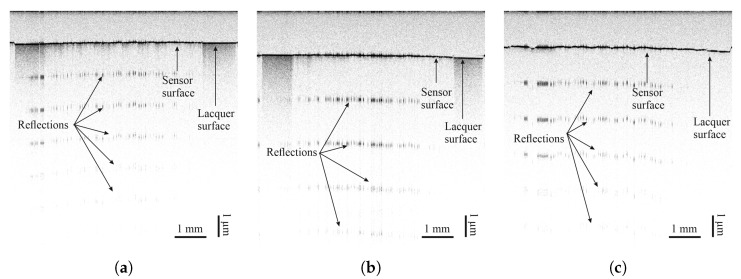
Images taken by optical coherence tomography (central wavelength of 830 nm) of airbrush-sprayed carbon nanotubes on (**a**) FACC, (**b**) TIGER, and (**c**) PRO.

**Figure 9:**
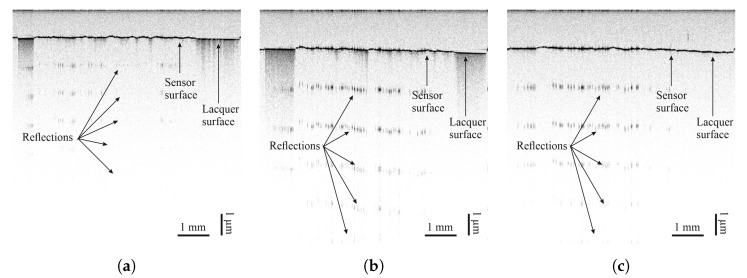
Images taken by optical coherence tomography (central wavelength of 830 nm) of inkjet-printed carbon nanotubes on (**a**) FACC, (**b**) TIGER, and (**c**) PRO.

**Figure 10:**
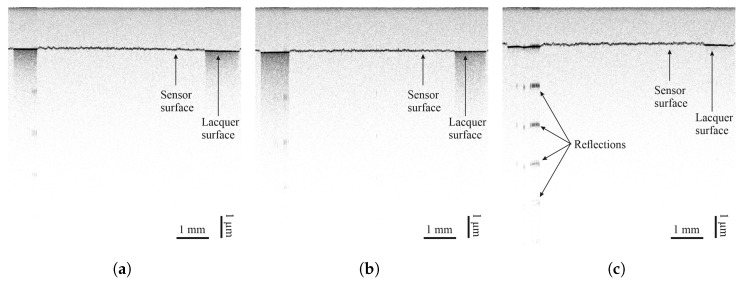
Images taken by optical coherence tomography (central wavelength of 830 nm) of carbon black on (**a**) FACC, (**b**) TIGER, and (**c**) PRO.

**Figure 11:**
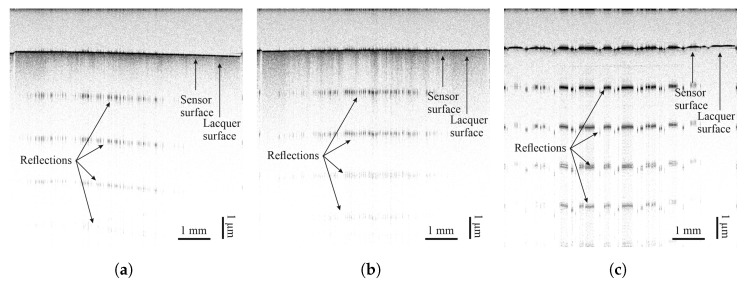
Images taken by optical coherence tomography (central wavelength of 830 nm) of PEDOT:PSS on (**a**) FACC, (**b**) TIGER, and (**c**) PRO.

Optical coherence tomography measurements of all sensor materials were carried out on all lacquers. Images can be seen in Figure 8, Figure 9, Figure 10 and Figure 11. Uneven distribution of the PEDOT:PSS sensor material can be seen from the inhomogeneous reflection of light from the lacquer underneath the sensor material since more of the light is absorbed in sensor-material-rich areas. It is more pronounced on the TIGER lacquer than on the FACC lacquer, coinciding with resistance measurements shown above. Evaluation of the sensor material distribution on PRO lacquer was inconclusive, since the lacquer absorbed most of the light emitted by the optical source. However, the lacquer’s surface appears to be rougher than the other lacquers. This is caused by the different application technique. The low-viscosity PEDOT:PSS can flow into the valleys of the lacquer, resulting in a limited number of conductive paths. Measurements of CNT-based sensors can been seen in Figure 8 and Figure 9. They indicate inhomogeneous sensor material distribution, although it is less pronounced and generally more opaque than PEDOT:PSS. Carbon black was evenly distributed, opaque on all surfaces, and formed a constant cross-section, as can been seen in Figure 10.

### 3.3. Piezoresistive Response Under Quasi-Static Stretch and Release Cycles

To determine the piezoresistive response of the sensors, all specimens were subjected to a quasi-static uniaxial tensile test with a cyclic loading pattern. These linear strain-and-release cycles followed a saw-tooth pattern at a frequency of 0.2 Hz for 50 cycles. The aluminium used for the PRO specimens turned out to be less stiff than the aluminium used for the FACC and TIGER specimens, resulting in higher strain levels. These blocks of 50 loading cycles were implemented before and after (hereafter referred to as pre- and post-fatigue, respectively) a $10^{6}$-cycle fatigue testing regime. This aims to detect any changes in the sensor’s response to strain after extensive loading and also allows for a detailed analysis of the sensor’s response to strain.

**Figure 12:**
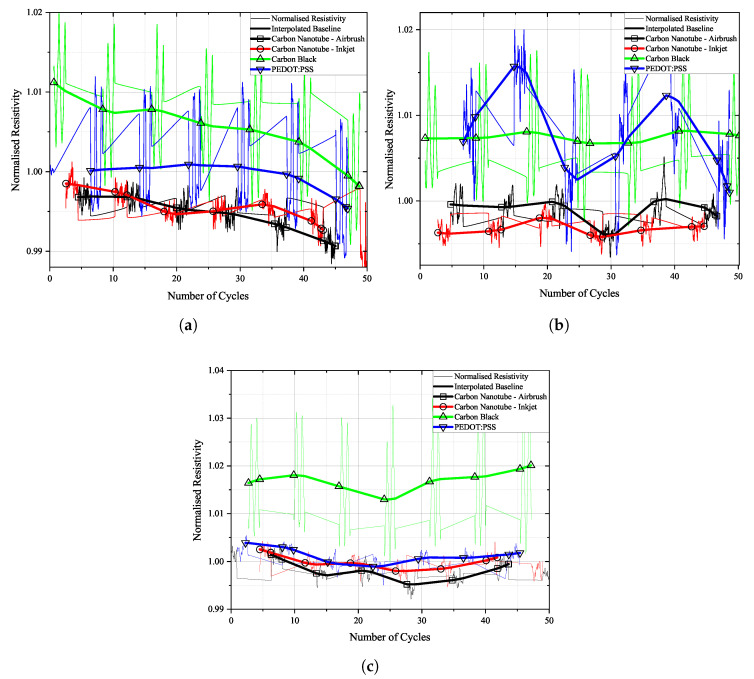
Normalised resistivity of sensors over time during the first 50 loading cycles on (**a**) FACC, (**b**) TIGER, and (**c**) PRO.

Sensors based on carbon allotropes can undergo significant resistivity changes during the first few loading cycles [4,34,35,36]. This change is attributed to the reorganisation of conductive particles. Temperature and relative humidity were constant during each of the 50 pre- and post-fatigue blocks. To allow for better evaluation and comparison of the sensor materials, normalised resistivity values were considered. The reference value $\rho_{0}$ was taken when the specimen was already clamped in the testing rig, but not yet under load. Results can be seen in Figure 12.

Surprisingly, a change in resistivity was observed only on the FACC specimen. Resistivity of all sensors dropped by about 1% over the course of the first 50 loading cycles. On the TIGER lacquer, resistivity was generally more stable for the carbon allotrope-based sensors. Noteworthy is the fluctuation in resistivity for the PEDOT:PSS sensor on the TIGER lacquer.

**Figure 13:**
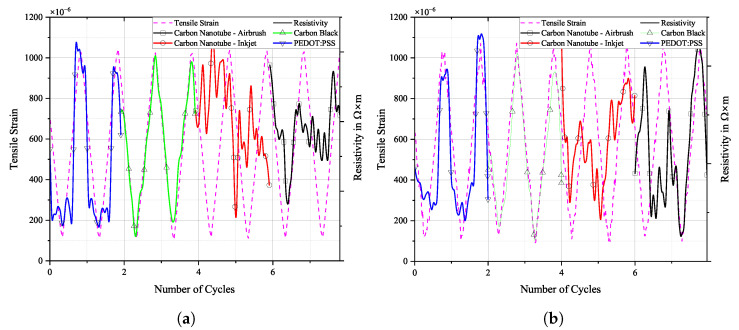
Tensile strain from DIC and resistivity of selected loading cycles over time, (**a**) before and (**b**) after fatigue loading on FACC specimen.

**Figure 14:**
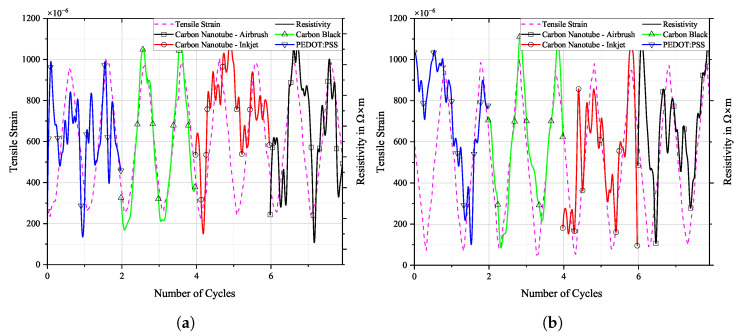
Tensile strain from DIC and resistivity of selected loading cycles over time, (**a**) before and (**b**) after fatigue loading on TIGER specimen.

**Figure 15:**
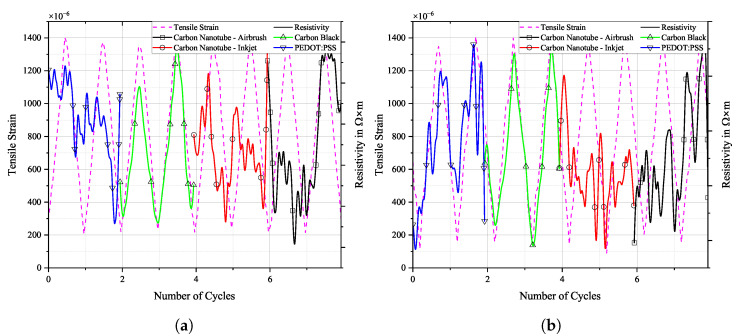
Tensile strain from DIC and resistivity of selected loading cycles over time, (**a**) before and (**b**) after fatigue loading on PRO specimen.

**Figure 16:**
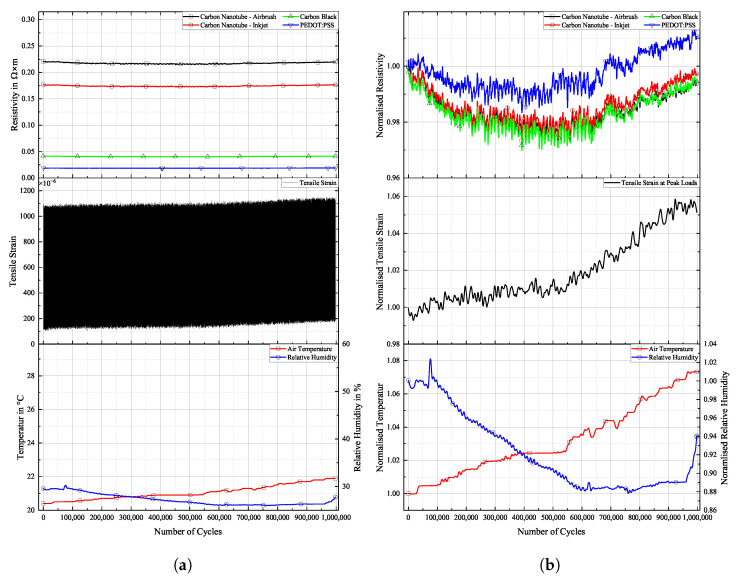
Long-term cyclic stability of sensor materials on FACC specimen over $10^{6}$ cycles. Top: individual resistivities; middle: tensile strain from DIC; bottom: air temperature and relative humidity; (**a**) measured data, (**b**) normalised dataset.

**Figure 17:**
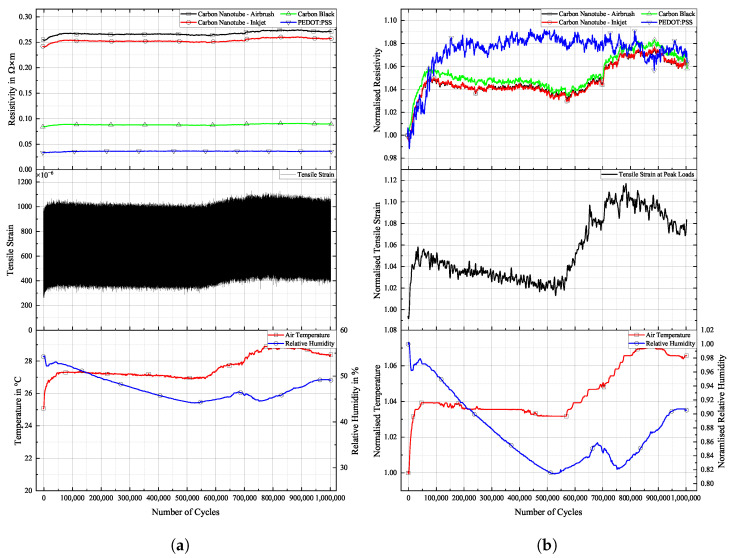
Long-term cyclic stability of sensor materials on TIGER specimen over $10^{6}$ cycles. Top: individual resistivities; middle: tensile strain from DIC; bottom: air temperature and relative humidity; (**a**) measured data, (**b**) normalised dataset.

**Figure 18:**
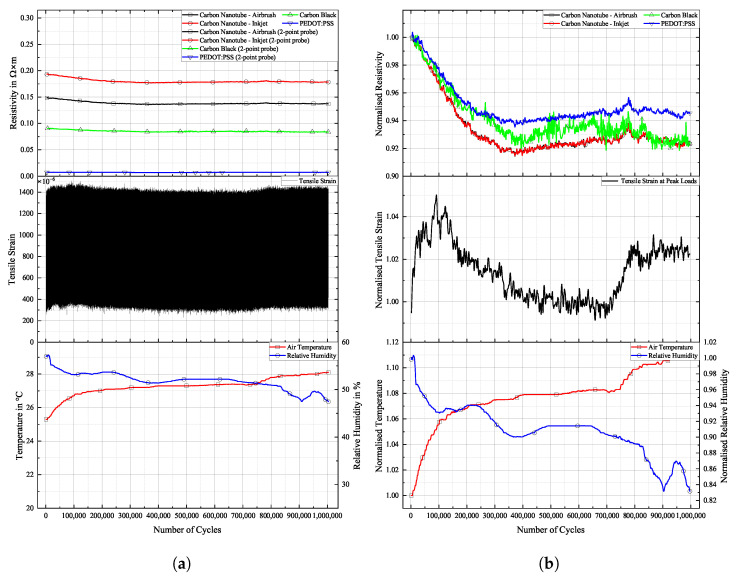
Long-term cyclic stability of sensor materials on PRO specimen over $10^{6}$ cycles. Top: individual resistivities; middle: tensile strain from DIC, bottom: air temperature and relative humidity; (**a**) measured data, (**b**) mormalised dataset.

Selected loading cycles and sensor responses can be seen in Figure 13, Figure 14 and Figure 15. Each sensor material’s signal was scaled individually on the right y-axis to allow for a more detailed depiction of the sensor’s change in resistivity when under strain. In reality, the resistivity values of the individual sensors are not equal. Therefore, the scale on the right does not show any scaling. Actual resistivity data can be seen in Figure 16, Figure 17 and Figure 18.

The change in resistivity of the CNT sensors is non-linear on all coatings/substrates regardless of application method and corresponds to the underlying tensile strain only in a few instances. A possible explanation for the non-linearity of both CNT-based sensors’ response could be that the strain levels were not high enough to stretch the entangled CNT bundles effectively. Thin-film sensors made from CNT composites are a popular material for the application as wearable sensors, and linear voltage responses have been reported. However, strain levels of interest in wearable sensors are considerably higher than for the application in aviation and may reach 100% [37]. Another factor could be the strong contribution of tunnelling resistance to the overall change in resistivity. Tunnelling resistance is generally considered to be non-linear [38]. The low CNT content of 0.3 wt.% results in only a small amount of CNTs overlapping extensively. This increases the contribution of tunnelling resistance to the overall change in resistivity. De Rijk et al. investigated the influence of CNT concentrations on sensor quality [35]. Not only did they find a two-phase response of the CNT sensor to strain, depending on strain level, but they also found that higher concentrations of CNT yield longer linear regions. Their conclusion was that high concentrations of CNT result in larger agglomerations that only disentangle at large strains. The sensor dimensions could also play a role. Zhao et al. investigated the behaviour of sprayed CNT sensors at a similar elongation level as in this study and found no non-linearities [19]. However, the proportion of MWCNT was 10 wt.%, and the sensors were twice as wide as in this study. Current but unpublished measurements at the time of writing support the assumption that sensor dimension has an influence. Sprayed CNT sensors of the same composition but 15 mm wide were stretched to about $1500\times 10^{-6}$ strain. A certain non-linearity was also observed, but it is significantly less pronounced than in the present study.

The alignment of CNTs can affect their strain sensing capabilities [39]. Although CNTs are oriented by inkjet printing in the printing direction [23,40], there are no obvious differences in the shape of change in resistivity compared to airbrush-sprayed CNTs.

Sensors made from carbon black depict deformation linearly and in a repeatable fashion. From Figure 12, it can be seen that the magnitude of the change in resistivity resulting from loading the sample is greatest for carbon black sensors on all substrates.

Sensors made of PEDOT:PSS show abrupt resistance changes on the FACC lacquer during loading and unloading. They form a plateau around the maximum and minimum elongation. The behaviour on the remaining samples is similar to that of CNT, i.e., non-linear and non-monotonic.

Cross-cut tests were carried out with an Erichsen cross-cut tester Model 295/I on the specimen according to ISO 2409 [41] and confirmed good bonding; characteristic values of 1 for the TIGER and PRO and 2 for the FACC samples could be determined. Thus, non-linearity as a result of insufficient bonding between aluminium and lacquer can be ruled out.

The sensitivity of a strain sensor is usually characterised by the gauge factor. It is a quantity for the change in electrical resistance to an applied elongation. Due to the non-monotonic change in the resistance of the CNT and PEDOT:PSS sensors, its determination is difficult. Sometimes during loading, a low strain level causes a large increase in resistance and vice versa, as can be seen in Figure 13, Figure 14 and Figure 15. Thus, the evaluation of the stain-sensing behaviour of the presented sensing materials based on a single point in the loading regime was deemed to be ineffective. Therefore, the correlation between the elongation of the specimen under stress and relief and the change in resistance of the individual sensors during the whole loading cycle is determined by means of the coefficient of determination $R^{2}$. The examination of the sensor’s change in resistivity as a reaction to the applied strain by the coefficient of determination $R^{2}$ allows for a continuous evaluation of the linearity of the sensor by just one value. The accuracy of the fit, which in this case is a linear k∗x+d-type, to the measurement data measured by $R^{2}$ can range from 0 to 1, with 1 indicating a perfect fit and thus a linear relationship of the sensor response to strain [42].

Linearity was evaluated for the full range of investigation in which the strain was examined in these pre- and post-fatigue tests. Resistivity was plotted over strain, a load cycle was selected, and a linear fit was calculated. A total of five cycles for each sensor material per quasi-static stretch and release sequence were evaluated. The results are shown in Table 1.

The response of CB to strain clearly exhibits a linear relationship with $R^{2}$ ranging from 0.88 to 0.97 with an average of 0.95 and 0.91 for pre- and post-fatigue loading, respectively, regardless of substrate. This indicates a slight deterioration of the sensor’s ability to adequately display the underlying strain.

CNT deposited by airbrush spraying exhibited a higher degree of linearity compared to CNTs deposited by inkjet printing, especially after long-term testing. However, all specimens displayed a highly non-linear behaviour. Both CNT sensor versions show a deterioration in performance, with the inkjet sensor deteriorating by more than 50% on average over all lacquers. Of all CNT sensors, inkjet-printed CNT on TIGER lacquer performed the worst with an $R^{2}$ of 0.07, but did not show any change over time. Interestingly enough, airbrush-sprayed CNTs on TIGER performed best with an $R^{2}$ of 0.36 before and 0.28 after fatigue loading.

The linearity of the voltage response to strain of PEDOT:PSS varies drastically when comparing substrates with values of $R^{2}$ ranging from 0.15 to 0.79. Interestingly, its linearity increased from pre- to post-fatigue measurements across all TIGER and PRO lacquers, but not on FACC.

The non-linearity displayed by all but carbon black sensors hinders their use for reliable strain sensing purposes. One could argue that even though the sensors do not show a linear response behaviour, a more advanced material characterisation with a quadratic or higher functional fit would better represent the characteristic and therefore could enable the application of non-linear sensors. Their unpredictable non-monotonic behaviour, however, prevents the inclusion of even a simple calibration process.

A decrease in a sensor’s sensitivity to strain after loading and unloading cycles is often observed [43]. This is attributed to the formation of additional conductive pathways when the surrounding matrix material deteriorates. However, an increase in sensor sensitivity has also been observed before [44]. Weak interaction between conductive particles leads to the irreversible formation of gaps. In contrast to the study mentioned here, this behaviour was not observed in the CB sensor, but for PEDOT:PSS.

### 3.4. Dynamic Durability of the Sensor Materials Under Extensive Fatigue Loading

Long-term performance stability and durability are of great importance for any type of sensor in structural health monitoring applications. This includes the sensor’s ability to maintain a stable electrical resistivity over time. Unforeseen changes in conductivity could lead to an over- or underestimation of prevailing strain states, be it a continuous state or just a single event. To investigate the sensor’s performance in regard to reliability and endurance, sensors were subjected to $10^{6}$ uninterrupted stretch-and-release cycles at constant load levels, as described in Section 2.5.

Figure 16, Figure 17 and Figure 18 show the cyclic response curves of the sensor materials on different specimens under stretching–releasing cycles over number of cycles and their respective normalised values. A reference value $\rho_{0}$ for normalisation was taken at the beginning of the experiment. Also displayed are tensile strain, room temperature, and humidity during testing.

The laboratory in which fatigue testing took place was subject to a natural fluctuation in temperature and relative humidity, with temperature levels being slightly elevated by the heat produced by the running testing machine. Changes in temperature over the course of the experiments resulted in a variation in tensile strain in the specimens. The positive thermal expansion of the aluminium caused the specimen to expand as temperature increased. This general increase in strain levels is apparent in the sensor’s change in resistivity. CNTs act as semi-conductors, thus providing a negative temperature coefficient [45]. Also, PEDOT:PSS shows a negative temperature coefficient [46]. Contrarily, CB networks display a positive temperature coefficient [47]. The magnitude of change in resistance for CNTs during testing is almost identical to that of CB. PEDOT:PSS displays a similar behaviour. The general behaviour of all sensors is that they follow more closely the changes in underlying strain than those of temperature. One can argue that a further characterisation of the temperature coefficient is mandatory to then perform a temperature correction of the sample, or at least to increase the signal stability.

All sensor materials dropped in resistivity during the first half of the experiment. It was a result of increasing temperatures in the vicinity of the specimen. It might also be an extension of the set-in effect seen in Figure 12a. Heinzlmeier et al. have recently recorded a set-in effect of carbon black that was present for the first 70,000 loading cycles [48]. This change in temperature was not large enough to have a significant effect on strain levels, as can be seen in Figure 16b. Changes in temperature and relative humidity cause a decrease in base resistivity levels of the sensor materials in operation as long as the underlying strain remains constant. This is peculiar since CB has a positive temperature coefficient; thus, increasing resistivity levels are expected. This hints at a possible influence of changing the relative humidity on the sensor. The resistivities of carbon allotrope sensors dropped by about 2%, while those of PEDOT:PSS dropped by 1%. After a plateau, the temperature again increased. This time, it showed an effect on strain levels, which increased accordingly. As soon as strain levels changed as a result of changing environmental conditions, sensor resistivities followed the changes in strain. All sensor materials displayed an increase in resistivity of similar magnitude. After 550,000 cycles until the end of the experiment, strain levels increased by 5%. During that time period, sensor resistivities increased by 2%.

Results for TIGER specimen can be seen in Figure 17.

Changes in resistivity for the carbon allotrope sensors are similar to what has been discussed above. Strain levels follow this change in temperature. Carbon allotrope-based sensors behaved as in the preceding experiment.

The sensor made from PEDOT:PSS behaved peculiarly. At first, resistivity increased as the strain level increased. After this, a short but steep initial increase occurred, and the strain levels dropped again as the air temperature started to decrease. But instead of following this trend, the resistivity of PEDOT:PSS kept increasing. Also, the second significant increase in strain levels during this experiment was not picked up by the PEDOT:PSS sensor. No single significant event of resistivity change can be seen in the graph above that would hint at damage to the sensor.

One possible explanation could be the partial detachment of PEDOT:PSS from the substrate. The combination of PEDOT:PSS and TIGER resulted in the lowest adhesion. Residual strain persisting within the sensor from production could cause the sensor to deform into a more stable state, as is often the case in polymer processing. The argument against this assumption is that PEDOT:PSS displays the highest linearity of change in resistivity to strain after long-term cyclic loading of all sensors tested. It even shows a remarkable improvement over the values compared to the initial $R^{2}$ measured.

Results for PRO specimen can be seen in Figure 18.

Similar to the experiment on FACC lacquer, resistivities of all sensor materials dropped extensively from the beginning of the experiment until 400,000 cycles. Apart from the initial sharp increase, all sensors displayed strain changes over the course of the experiment. The decline in strain levels of 5% led to a drop in resistivity of 6% and 8% for the CNT sensors. CB lies in between. A steep incline in strain levels caused by rising temperatures starting after 700,000 cycles led to a general rise in resistivity in all sensors. PEDOT:PSS shows similar behaviour to that of CNT sensors but at a lower resistivity. The percentile drop in resistivity over time due to change in strain is not as pronounced. CB again is more sensitive to the strain changes.

## 4. Conclusions

In this study, we have applied three piezoresistive materials, two of which are based on allotropes of carbon and one PEDOT:PSS, using three different application methods onto three disparate lacquer systems. These lacquers are polyurethane- and polyester-based, and were applied in wet conditions and powder-coated, respectively. A third lacquer variant is acrylate-based and was applied by inkjet printing.

The sensor–lacquer system was characterised for adhesiveness. Contact angle measurements of the sensor materials and the lacquers were made. Based on these measurements, work of adhesion between the sensor materials and lacquers was evaluated. Carbon black showed best overall adhesive properties. The surface displaying the best adhesive properties was found to be PRO, followed closely by FACC. Sufficient adhesion of all sensor materials to all lacquers was shown by the tape adhesion test.

Furthermore, the distribution of sensor materials was analysed. Resistance measurements whilst shortening the sensor length showed a homogeneous distribution of CNT and CB on all lacquers. Measurement of PEDOT:PSS hinted at an in-homogeneous distribution, especially pronounced on TIGER lacquer. This was confirmed by optical coherence tomography. Although both carbon nanotube variants also displayed an uneven distribution, it was not severe enough to impact their electrical properties.

The sensor material’s piezoresistive behaviour under strain was tested and evaluated by quasi-static stretch-and-release tests. A detailed analysis was conducted before and after an extensive fatigue loading regime. Non-linear effects were detected for sensors based on carbon nanotubes and PEDOT:PSS. The non-linearity intensified for all carbon allotrope-based sensors after long-term loading, while for PEDOT:PSS, the linearity increased significantly. Carbon black showed the best strain sensing performance, both in linearity and presumed sensitivity. But the high percolation threshold and therefore high volume percent of material needed to produce a conductive sensor prohibit it from being used as the conductive filler in low-viscosity, sprayable inks. Airbrush spraying of sensor materials is a fast and easy way of covering large and curved areas for global strain sensing applications. The mixing of CNT and CB into one applicable ink is a promising approach to eliminate these drawbacks [49,50].

The sensors’ stability and durability were evaluated by subjecting them to $10^{6}$ cycles of constant cyclic tensile loading. Temperature and humidity fluctuations during cyclic testing resulted in inconsistent strain levels in the specimens. While this is not ideal for scientific evaluations, it reflects real-world conditions. Temperature coefficient corrections in combination with a near-strain-gauge temperature measurement might be needed to stabilise the sensor signal response when using the sensor materials in situations where environmental conditions are not constant. Another promising approach to temperature compensation is the combination of conductive materials with different temperature coefficients within one ink. CNTs and graphene exhibit opposing temperature coefficients. Thus, by choosing an adequate mixing ratio, their temperature sensitivities will cancel each other out and create a self-temperature-compensated thin-film sensor [45,51]. In order to compensate for humidity changes, a protective top could be applied onto the sensors, which would also protect the sensors from corrosion or scratches. Enser et al. applied a protective organic coating to a sensor made from carbon black [4]. They reported that after the application of the topcoat, a hysteresis was no longer existent, and the sensor’s behaviour was described as being linear. But at the same time, the gauge factor dropped by almost 50 %. How topcoats affect the strain sensing capabilities of sensors made from sprayable inks is a subject of future investigation. Despite the temperature and humidity fluctuating, all sensor materials did follow the underlying strain changes with the exception of PEDOT:PSS on TIGER.

## Figures and Tables

**Table 1:** Coefficient of determination $R^{2}$ for sensor materials categorised by lacquer systems before and after $10^{6}$ cycles of loading. The closer values are to 1, the better the linear fit.

	CNT—Airbrush		CNT—Inkjet		CB		PEDOT:PSS	
	Pre-Fatigue	Post-Fatigue	Pre-Fatigue	Post-Fatigue	Pre-Fatigue	Post-Fatigue	Pre-Fatigue	Post-Fatigue
FACC	0.29	0.22	0.24	0.06	0.96	0.94	0.79	0.71
TIGER	0.36	0.28	0.07	0.07	0.92	0.88	0.18	0.74
PRO	0.21	0.21	0.31	0.14	0.97	0.91	0.15	0.43
Average	0.28	0.24	0.21	0.09	0.95	0.91	0.37	0.63

## Data Availability

The raw data supporting the conclusions of this article will be made available by the authors on request.
